# Evaluation of Some Potentially Toxic Elements and Associated Ecological and Health Risks in Topsoil Samples Adjacent to an Industrial Zone in Turkey

**DOI:** 10.1021/acsomega.3c07638

**Published:** 2024-10-14

**Authors:** Aydan Altıkulaç, Şeref Turhan

**Affiliations:** † Ula Ali Koçman Vocational School, Muğla Sıtkı Koçman University, 48640 Ula, Muğla, Turkey; ‡ Department of Physics, Faculty of Science, Kastamonu University, 37150 Kastamonu, Turkey

## Abstract

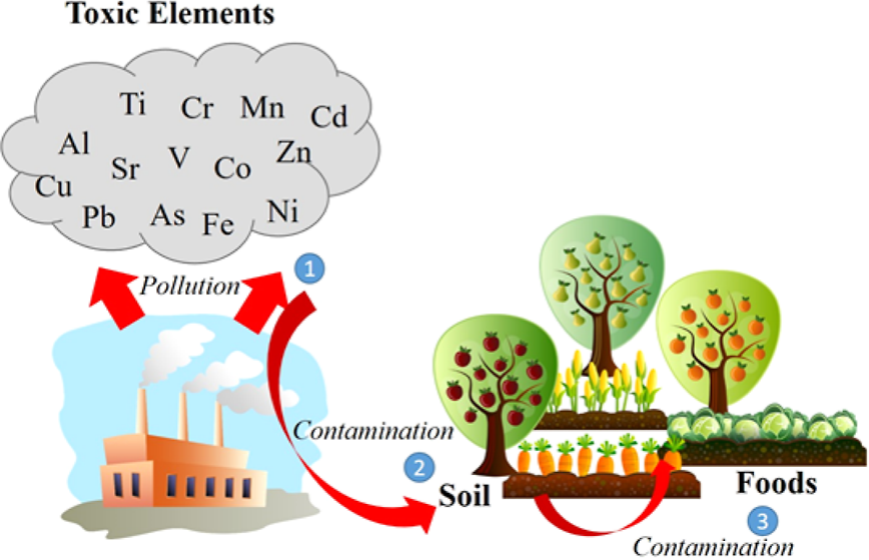

Potentially toxic element (PTE) pollution as a result of industrial activities remains a global problem that poses serious threats to human and ecological health. PTEs (Al, Fe, Ti, Mn, V, Zn, Cu, Cr, Ni, Pb, Co, and As) are metals or metalloids with biological toxicity. This study analyzed the concentrations of these PTEs and the physicochemical properties of topsoil samples collected from areas near industrial districts in the Samsun province of Turkey to evaluate ecological and health risks, estimating various indexes. The average concentrations of Al, Fe, Ti, Mn, V, Zn, Cu, Cr, Ni, Pb, Co, and As analyzed in 23 topsoil samples by energy-dispersive X-ray fluorescence spectrometry were found as 93,822, 82,410, 6623, 1642, 406, 278, 207, 149, 78, 68, 32, and 10 mg/kg dry weight, respectively. Zn, Cu, Cr, Ni, Pb, and Co levels exceed the maximum contaminant levels in the Turkish Regulation on the Control of Soil Pollution. The average pH values, organic matter, total organic carbon, and nitrogen measured in soil samples were 7.14, 6.11, 0.96, and 0.04%, respectively. The ecological and health evaluation reveals that the studied area is polluted with V, Cu, Zn, As, Ni, and Pb, which may pose a risk to people living in settlements near the industrial district.

## 1 Introduction

The rapid growth of population, urbanization, economy, and industrialization causes more pollution of the environment and disruption of ecological balance, which continues to be an essential global problem that is becoming increasingly difficult to solve.^1,2^ Environmental pollutants are toxic substances originating from industrial activities such as fossil fuel burning (thermal power plants, iron and steel factories, cement industries, etc.), smelters, mining activities, inorganic fertilizer facilities, agrochemicals, and electronics production, etc.^2−5^ Environmental pollutants, which often contain potentially toxic elements (PTEs) and inorganic chemicals, cause dangerous problems for abiotic environments such as soil, water, and air and biotic communities including all living organisms such as humans, animals, and plants, and thus, these pollutants significantly disrupt the structure and function of the entire ecosystem.^1^

Soil is a crucial constituent of the ecosystem, and it is a necessary environment for energy exchange between plants and the natural ambient and material cycling.^6^ Soil is vital in sustaining human life, providing approximately 96% of the food humans need.^7,8^ However, the soil is the permeable surface of the Earth and is prone to accumulation with environmental pollutants.^4^ Pollution of the soil with PTEs changes the physicochemical properties of the soil and reduces its fertility.^8^ The intense accumulation of these PTEs in the soil contaminates the food chain and directly or indirectly threatens human health. PTEs are generally classified as carcinogens and noncarcinogens. Carcinogenic PTEs contain As, Hg, Cd, Pb, Cr, Ni, etc.^9^ Noncarcinogenic PTEs such as Cu, Fe, Co, Zn, and Mn have functional roles and are essential for human biochemical and physiological functions.^10^ Long-term human exposure to these PTEs by ingestion, inhalation, and dermal contact can lead to carcinogenic or noncarcinogenic diseases such as genetic disorders, tumors, cognitive and temperamental decline, liver and kidney damage, heart disease, nephrotic syndrome, and neurotoxicity.^4,11,12^ Therefore, it is crucial to know the level of toxic element contamination or pollution in the soil to evaluate the ecological and health risks arising from toxic elements due to natural or human activities.

In recent years, many studies have been conducted to evaluate soil contamination with PTEs or heavy metals (HMs) in industrial areas worldwide.^4,5,8,13−28^ However, few studies were published on evaluating the pollution of industrial soils with PTEs,^29−32^ while several studies were performed to investigate the contamination of soil samples with PTEs or HMs due to traffic emissions in Turkey.^33−39^ Çubukçu and Tüysüz^29^ analyzed the concentrations of Cu, Zn, Pb, Fe, Mn, Cd, and S in soil, plant, and water samples collected from around industrial areas located in Tekkeköy (Samsun province) using inductively coupled plasma-atomic emission spectrometry. Yaylalı-Abanuz^30^ analyzed the concentrations of As, Cr, Cd, Hg, Cu, Mn, Zn, and Pb in surface soil samples collected from large organized industrial zones in the Gebze district of Kocaeli province using inductively coupled plasma–mass spectrometry. Also, they calculated ecological risk indices to assess soil contamination. Yatkın and Bayram^31^ determined the levels of Al, Ca, Fe, Mg, K, Pb, Zn, Cd, etc., in soil samples collected from industrial, urban, rural, and agricultural sites of İzmir province to investigate the modification in the elemental composition due to anthropogenic activities. Koz et al.^32^ analyzed the concentrations of Al, V, Cr, Mn, Fe, Ni, Cu, Zn, As, and Pb in moss and soil samples collected in the neighborhoods of the Murgul copper mining in Artvin province using energy-dispersive X-ray fluorescence (EDXRF) spectrometry. According to the literature review, there is no detailed study yet on the ecological and health risk evaluation that may be caused by soil contamination in the Tekkeköy (Samsun) industrial zone. Tekkeköy industrial zone (TIZ), one of Turkey’s important industrial regions, is located in the Tekkeköy district of Samsun, located in the Central Black Sea region. TIZ includes the phosphate fertilizer production plant (PFP), phosphogypsum storage area (PSA), iron and steel factory (ISF), copper smelting plant (CSP), and organized industry facilities. The facilities in the TIZ cause environmental pollution, such as air, soil, and water pollution. Having all these in mind, in this study, (1) the concentrations of major-minor oxides and PTEs (Al, Fe, Ti, Mn, V, Zn, Cu, Cr, Ni, Pb, Co, and As) in 23 surface soil samples collected from areas close to the PFP were analyzed using an EDXRF spectrometer, and the average values of PTEs were compared with the limit values in the Regulation on Control of Soil Pollution of Turkey,^40^ (2) pollution indicators [single pollution index (PI), pollution load index (PLI), geo-accumulation index, and enrichment factor (EF)] were calculated to evaluate the ecological risk due to PTEs, and (3) average daily dose (ADD), health quotient, hazard index (HI), incremental lifetime cancer risk (ILCR), and cancer risk indexes were estimated to evaluate the carcinogenic and noncarcinogenic health risks of PTEs via ingestion.

## 2 Materials and Methods

### 2.1 Study Area

Tekkeköy, where the industry is located, is a district of Samsun province located in the Central Black Sea region of Turkey. Tekkeköy district, with an area of 362 km^2^, is between 41°01′20″ and 41°15′50″ N latitudes and 36°21′30″ and 36°35′30″ E longitudes. According to the 2022 census, the population of the district is 56,318. Tekkeköy is located at the northwest end of the northern Anatolian mountains, formed in the Tertiary and established on a fertile plain to the west of the Yeşilırmak delta. Tekkeköy is located between Eocene-aged Tekkeköy Formation and Quaternary-aged alluvium.^29^ Tekkeköy Formation consists of basalt, andesite, agglomerate, sandstone, siltstone, conglomerate, and tuffite. TIZ is located between 41°04′30″ and 41°13′40″ N latitudes and 36°26′10″ and 36°27′25″ E longitudes. The soil structure of TIZ consists of clayey, sandy-clayey, and silty soils composed of alluviums carried by rivers.^29^ The industrial facilities in the TIZ, established on an area of approximately 10 km^2^, constitute the primary source of environmental pollution. PFP, PSA, ISF, and CSP are the most important polluting sources. The PFP includes a sulfuric acid facility, two phosphoric acid facilities, fertilizer facilities, and a phosphogypsum waste area. The PFP has been operating for nearly 50 years and has a fertilizer production capacity of 575,000 tons/year. Approximately 3000 tons of phosphogypsum produced daily is transported to the waste area close to the facility and the Black Sea without recycling and processing.

### 2.2 Collection and Preparation of the Soil Samples

**Figure 1:**
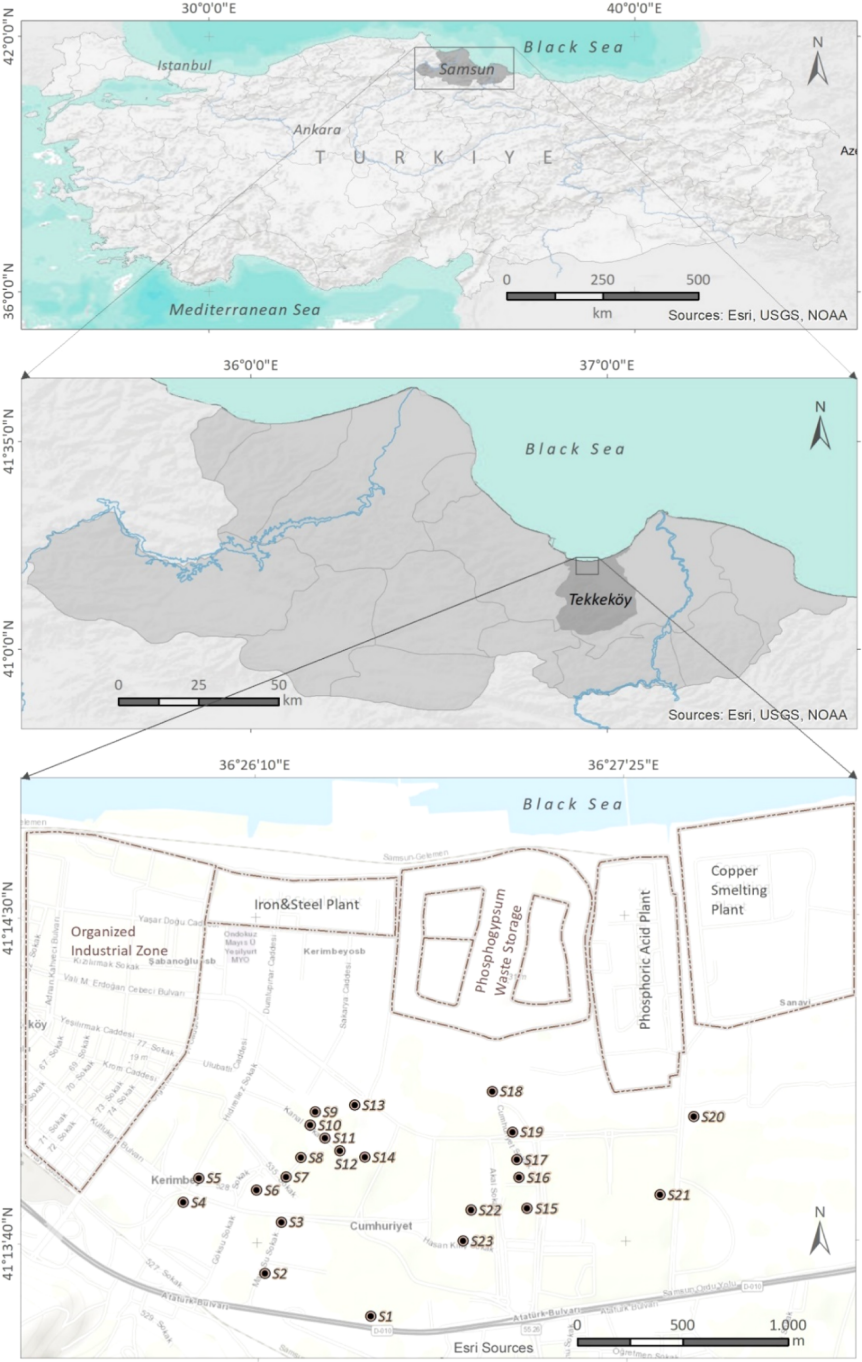
Locations of the sampling sites.

23 topsoil samples weighing approximately 0.5–1 kg were randomly collected from locations shown in Figure 1 to represent the sample study, and a uniform distance between the sampling points was not used. Soil samples were collected using an earth auger (made of stainless steel and 76 × 102 mm) and soil samplers (made of steel and 279 × 360 mm). The reason for choosing topsoil sampling (0–10 cm) is that the top surface layer (above 25 cm) is usually affected by toxic metals where the roots of plants or crops are located.^41^ Since PFP constitutes the largest source of environmental pollution in TIZ, as shown in Figure 1, most of the samples were collected from places close to PSA. The samples brought to the sample preparation laboratory in plastic bags were kept in an atmospheric environment for a while and then dried in the oven at 105 °C for 3 h. The dried samples were then ground into powders for the calibrated powder geometry in the EDXRF spectrometer.

### 2.3 Measurement of the Physicochemical Properties of the Soil Samples

The pH value was measured for soil and distilled water in the ratio (1:2.5) by using a Milwaukee portable pH meter (MW101 model) for 1 h in solution. The amount of organic matter (OM) in the samples was estimated using the loss of ignition method. It was measured by burning 5–10 g of each dried sample in a muffle furnace (Carbolite ELF, 11/14B model) at 550 °C for 5 h. The bulk density of each sample was determined by the weight loss after drying the undisturbed soil cores. The total organic carbon (TOC) and nitrogen (N) were measured in a CHN/S elementary analyzer (EuroVector, EA 3000 model) according to the dry combustion method.

### 2.4 Analysis of Potentially Toxic Elements

The analysis of PTEs in the soil samples was carried out using an EDXRF spectrometer (Spectro Xepos, Ametek) with a thick binary Pd/Co alloy anode X-ray tube (50 kV, 60 W). Detailed knowledge of the EDXRF spectrometer was presented in previously published studies.^3,42,43^ The spectrometer uses advanced techniques of calibration, such as “nonstandard” calibration, often based on the fundamental parameters method.^3^ TurboQuant II software quickly and accurately analyzes any unknown powder, solid, or liquid sample.^3^ Soil-certified reference material (NIST SRM 2709) was used for the quality assurance of the spectrometer.^3^ Each soil sample was placed in the automatic sampler and counted once for 2 h, and the analysis processes were completed. The overall uncertainties (%) of Al, Fe, Ti, Mn, V, Zn, Cu, Cr, Ni, Pb, Co, and As were found as 0.1, 0.1, 0.2, 0.2, 1.4, 0.6, 1.0, 1.1, 1.4, 1.6, 7.8, and 5.2.

### 2.5 Evaluation of Ecological Risk

Ecological risk evaluation is an important analysis in which the effects and extent of pollution of PTE in the soil can be estimated, which may also be related to human health.^25^ In this study, ecological risk evaluation was carried out with the help of the following indexes. The single PI can determine which PTE represents the highest threat to a soil environment and is characterized as the PTE concentration in a sample relative to the geological background level.^44,45^ The PI is also required to estimate the PLI. The PI is estimated by using the following equation^44,45^
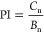
1where *C*_n_ is the concentration of PTE analyzed in the soil samples, and *B*_n_ is the geochemical background value of the corresponding PTE. In the PI calculations, the Earth’s crust average (ECA) values given in the study by Yaroshevsky^46^ were taken as the geochemical background values of the PTEs analyzed in the soil samples. Based on PI values, pollution is classified as follows: PI < 1: absent; 1 < PI < 2: low; 2 < PI < 3: moderate; 3 < PI < 5: strong, and PI > 5: very strong.^44^ The PLI is used to evaluate the overall PTE contamination degree in the soil.^47^ This index provides an easy way to demonstrate the deterioration of soil conditions due to PTE accumulation. It is calculated as the geometric average of PI based on the following formula^44^
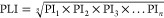
2where PI is the single PI in eq 1, and n is the number of analyzed PTEs. Based on PLI values, pollution degree is categorized as follows: PLI < 1: perfection; PLI = 1: only baseline pollution levels; and PLI > 1: deterioration of soil quality.^44^ The geo-accumulation index (*I*_geo_) enables the evaluation of soil contamination with PTE by comparing preindustrial and current PTE concentrations.^48^*I*_geo_ reports the classification of PTE pollution in the soils and is estimated using the following equation^3,25^
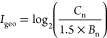
3Factor 1.5 accounts for natural fluctuations because of possible differences in the reference values and minimal anthropogenic influences.^3,25^ Based on *I*_geo_ values, contamination is classified as follows: *I*_geo_ ≤ 0: uncontaminated; 0 < *I*_geo_ < 1: uncontaminated to moderately contaminated; 1 ≤ *I*_geo_ < 2: moderately contaminated; 2 ≤ *I*_geo_ < 3: moderately to heavily contaminated; 3 ≤ *I*_geo_ < 4: heavily contaminated; 4 ≤ *I*_geo_ < 5: heavily to extremely contaminated; and *I*_geo_ ≥ 5: extremely contaminated. The EF is an index used to evaluate the possible effect of anthropogenic activity on PTE concentration in soil samples.^44,49^ To determine the expected impact of anthropogenesis on PTE concentrations in the soil samples, the content of PTEs characterized by low occurrence variability is used as a ref (44). Reference elements are usually Ca, Al, Sc, Mn, Ti, Sr, Fe, and Zr.^3,44,49^ EF is calculated using the following formula^3,44^
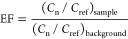
4where *C*_n_ and *C*_ref_ are the concentration of PTE and reference element in the soil sample and geochemical background, respectively. In the EF calculations, Zr was taken as the reference element. Zr was considered practically not released by human activities because Zr has no remarkable anthropogenic source and is primarily found in the weather-resistant heavy mineral zircon.^3,49^ Based on the EF values, contamination is classified as follows: EF < 2: deficiency to minimal; 2 ≤ EF < 5: moderate enrichment; 5 ≤ EF < 20: significant enrichment; 20 ≤ EF < 40: very high enrichment; and EF ≥ 40: extremely high enrichment.^3^

### 2.6 Evaluation of Health Risk

Potential health risk evaluation is the process of estimating the nature and probability of adverse health effects on people who may be exposed to hazards in polluted or contaminated environmental media.^50^ Health risks of pollutants often include carcinogens and noncarcinogenic risks. This study accepted the potential health risk evaluation model recommended by the USEPA. The ingestion of soil is a potential way of exposure to environmental chemicals for adults and children.^51^ Adults living near TIZ may be exposed to soil and dust from hobby gardening in front of their homes or while traveling to and from work areas.

The cumulative noncarcinogenic and carcinogenic risks (CR) are represented by HI and CR, respectively. The ADD was calculated for adults as follows^25,52^
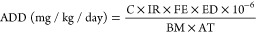
5where *C* is the PTE concentration in sepiolite samples (mg/kg); IR is the ingestion rate of soil and dust (100 mg/day) given for adults in the final report prepared by USEPA;^51^ ED is the average exposure duration of adults (70 y); FE is the frequency of exposure (365 day/y); BM is the average body weight of adults (77 kg); and AT is the average exposure time (79 y × 365 day/y). Then, based on the ADD value, the hazard quotient (HQ) for the noncarcinogenic HI was estimated using the following equation^25,52^
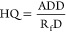
6
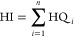
7where R_f_D is the PTE oral reference dose and 1, 2, ..., *n* are the individual PTE in the sepiolite samples. The RfD value for Al, V, Cr, Mn, Fe, Co, Ni, Cu, Zn, As, and Pb was taken as 0.1, 0.009, 0.003, 0.14, 0.007, 0.0003, 0.02, 0.005, 0.3, 0.0003, and 0.0035 mg/kg/day, respectively.^53^ For noncarcinogenic risk, HQ and HI > 1 values indicate high potential adverse health effects.^25,52^

Based on the ADD value, the ILCR for CR was calculated for Cr, Ni, As, and Pb by using the following equation^25,52^
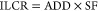
8
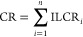
9where SF is the slope factor and is taken as 0.5, 1.7, 1.5, and 0.0085 per mg/kg/day for Cr, Ni, As, and Pb, respectively.^53^ Cancer risk levels are evaluated in four classes as follows: CR ≤ 10^–6^: no risk; 10^–6^ < CR < 10^–5^: acceptable risk; 10^–5^ < CR < 10^–4^: low priority risk; and CR ≥ 10^–4^: high priority and unacceptable risk.^54^

## 3 Results and Discussion

### 3.1 Major and Minor Oxides of the Soil Samples

**Figure 2:**
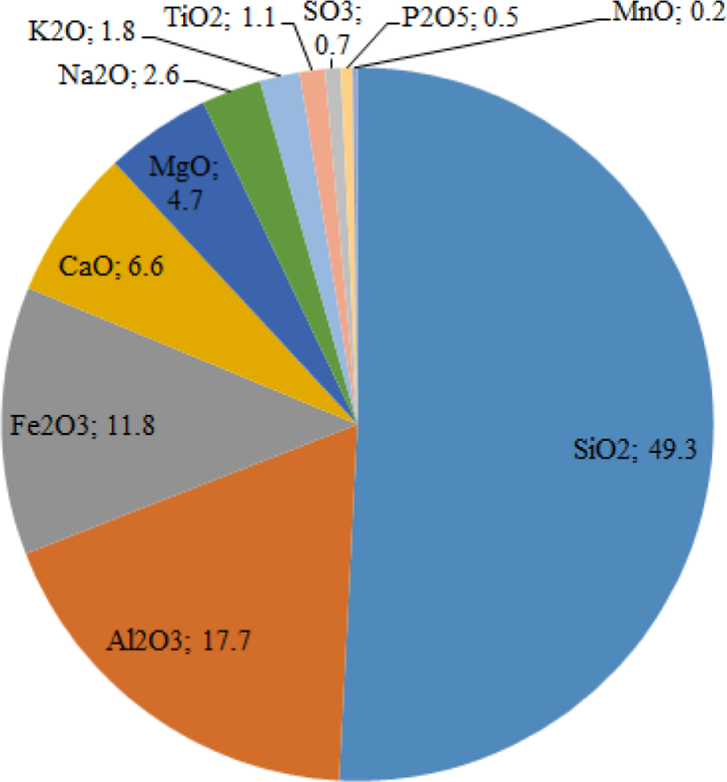
Major and mineral oxides of the soil samples in percentage.

The distribution of major and minor oxides analyzed in the soil samples is shown in Figure 2. According to the average concentration (wt %), the major-minor oxides in the soil samples were ordered as SiO_2_ (49.3) > Al_2_O_3_ (17.7) > Fe_2_O_3_ (11.8) > CaO (6.6) > MgO (4.7) > Na_2_O (2.6) > K_2_O (1.8) > TiO_2_ (1.1) > SO_3_ (0.7) > P_2_O_5_ (0.5) > MnO (0.2). The concentrations of SiO_2_, Al_2_O_3_, Fe_2_O_3_, CaO, MgO, Na_2_O, K_2_O, TiO_2_, SO_3_, P_2_O_5_, and MnO varied from 32.8 to 56.3, 8.1 to 24.2, 8.3 to 21.4, 2.1 to 14.1, 3.1 to 6.3, 1.8 to 4.1, 0.5 to 2.9, 0.8 to 2.0, 0.1 to 5.4, 0.2 to 1.0, and 0.2 to 0.3%, respectively. As shown in Figure 2, the soils (approximately 96%) mainly consist of major oxides. All minor oxides (SO_3_, P_2_O_5_, and MnO) constitute 1.4% of the soil. The average concentrations of Al_2_O_3_, Fe_2_O_3_, K_2_O, TiO_2_, SO_3_, and P_2_O_5_ are higher than the average concentration of 15.9, 1.1, 1.1, 1.0, 0.05, and 0.2% in the Earth’s crust,^46^ respectively.

### 3.2 Physicochemical Properties of the Soil Samples

The values of physicochemical parameters measured for the investigated soil samples are listed in Table 1. The values of pH, OM, salinity, and bulk density varied from 5.74 to 7.86 with an average of 7.14, 2.10 to 14.45% with an average of 6.11%, 0.22 to 0.05 ppt with an average of 0.38 ppt, and 0.99 to 1.82 g/cm^3^ with an average of 1.33 g/cm^3^, respectively. The average TOC and *N* values were 0.96 and 0.04%, respectively. The average pH value of the soil samples around the phosphogypsum deposit was very close to neutral.

**Table 1:** Various Physicochemical Properties of the Topsoil Samples

sample code	pH	salinity (ppt)	OM (%)	bulk density (g/cm^3^)
S1	7.03	0.34	6.54	1.26
S2	7.42	0.20	7.85	1.30
S3	7.08	0.30	9.59	0.99
S4	7.15	0.38	4.86	1.26
S5	6.28	0.32	6.60	1.32
S6	7.20	0.12	6.50	1.34
S7	6.58	0.22	5.39	1.29
S8	7.23	0.21	5.85	1.41
S9	7.30	0.22	2.31	1.42
S10	7.30	0.18	2.10	1.48
S11	7.78	0.33	6.69	1.11
S12	7.64	0.24	4.21	1.47
S13	6.78	0.11	3.95	1.48
S14	7.68	0.05	3.01	1.82
S15	7.26	0.15	4.61	1.04
S16	7.85	0.06	5.31	1.65
S17	7.86	0.15	9.06	1.20
S18	7.62	0.12	5.02	1.53
S19	6.95	0.37	10.40	1.18
S20	6.43	0.25	4.13	1.18
S21	7.09	0.12	5.94	1.36
S22	5.74	0.35	14.45	1.09
S23	6.99	0.23	6.14	1.46
average	7.14	0.22	6.11	1.33
min	5.74	0.05	2.10	0.99
max	7.86	0.38	14.45	1.82

## 4 PTE Concentration

**Figure 3:**
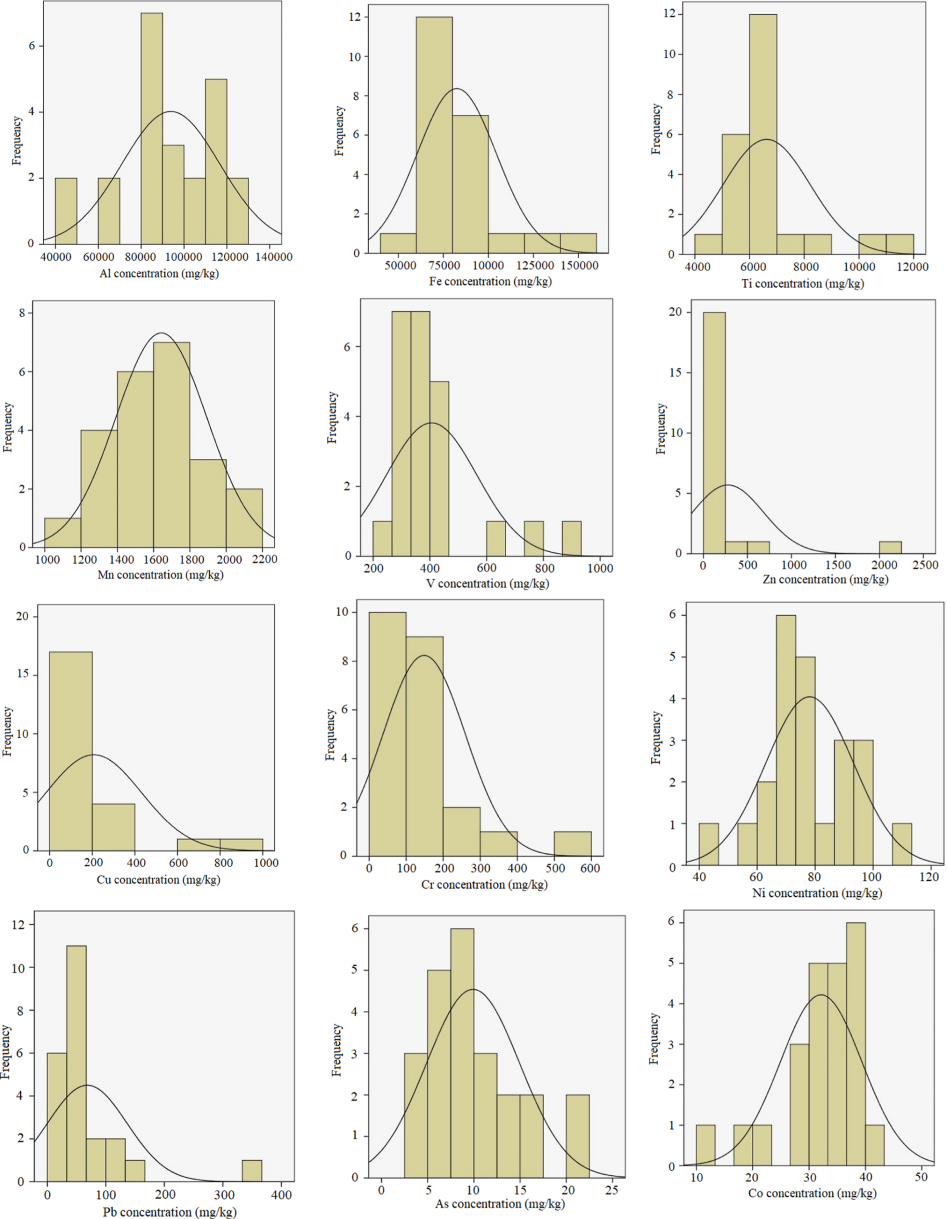
Frequency distributions of PTE concentrations in soil samples. Furthermore, the correlation analysis also showed potential associations in the physicochemical properties of the topsoil samples (Figure 4). This investigation presented statistically significant negative correlations at a significance level of *p* < 0.01. The correlations, which exhibited statistical significance at the *p* < 0.01 level, were observed between salinity—density (−0.64) and OM—density (−0.59).

The PTE concentration [in mg/kg dry weight (dw)] of each soil sample and some descriptive statistics on the concentrations of PTEs analyzed in all soil samples are given in Table 2. The frequency distributions of the PTE concentrations are shown in Figure 3. From Table 2, the average PTE concentrations follow this order: Al > Fe > Ti > Mn > V > Zn > Cu > Cr > Ni > Pb > Co > As.

**Table 2:** Concentration of PTEs and Some Descriptive Statistics^a^

sample code	the concentration of PTE (mg/kgdw)											
	Al	Ti	V	Cr	Mn	Fe	Co	Ni	Cu	Zn	As	Pb
S1	113,500	6280	374	82	1577	83,150	38	71	59	116	3	26
S2	94,550	6328	331	94	1420	76,090	34	81	172	169	7	51
S3	121,800	5176	279	73	1243	61,810	17	64	319	132	8	41
S4	89,260	6010	356	132	1787	76,820	36	79	135	198	7	53
S5	114,300	5353	306	105	1395	73,340	40	71	109	143	3	32
S6	110,300	6063	353	80	1597	81,310	39	72	145	196	6	48
S7	106,600	7736	464	87	2020	94,880	37	79	166	232	12	95
S8	119,700	5943	334	103	1374	69,220	31	57	102	129	7	56
S9	88,380	8408	605	121	1818	105,600	27	69	132	230	8	99
S10	62,540	10,100	776	533	1983	136,400	35	95	124	233	16	141
S11	99,130	6207	412	147	1574	83,350	33	111	355	650	22	338
S12	87,340	6856	428	194	1787	82,580	20	97	981	2042	14	130
S13	116,800	6594	418	59	1651	76,620	27	44	107	124	7	44
S14	42,750	11,800	906	343	2169	149,700	42	92	33	109	9	20
S15	84,980	6586	397	167	1730	77,690	32	98	218	173	13	41
S16	49,550	6346	454	266	1636	81,380	39	78	45	83	8	18
S17	92,770	6201	282	74	1515	68,060	32	74	64	101	4	30
S18	85,230	4796	273	266	1168	57,700	13	73	736	174	20	54
S19	88,340	5529	274	91	1564	66,350	36	72	130	216	10	43
S20	109,200	6482	397	64	1694	80,910	32	62	78	147	8	29
S21	63,240	5439	317	126	1790	71,820	30	91	138	401	10	37
S22	128,300	5558	235	68	1377	61,520	35	73	263	173	12	103
S23	89,350	6548	366	143	1908	79,130	37	93	157	220	15	39
average	93,822	6623	406	149	1642	82,410	32	78	207	278	10	68
median	92,770	6280	366	105	1636	77,690	34	74	135	173	8	44
SD	22,817	1595	160	111	251	21,933	7	15	224	402	5	68
SE	4758	333	33	23	52	4573	2	3	47	84	1	14
kurtosis	0.01	4.8	4.2	5.8	–0.3	4.3	1.4	0.2	7.1	18.7	0.5	11.6
skewness	–0.7	2.1	2.0	2.3	0.1	2.0	–1.3	0.1	2.6	4.2	0.9	3.1
min	42,750	4796	235	59	1168	57,700	13	44	33	83	3	18
max	128,300	11,800	906	533	2169	149,700	42	111	981	2042	22	338

a SD: standard deviation; SE: standard error.

It can be seen from Figure 3 that the concentration distributions of Fe, V, Zn, Cu, Cr, and Pb exhibit a log–normal distribution, while Al, Ti, Ni, Co, and As have a non-normal distribution. The Mn concentration distribution fits the normal distribution.

The Al concentrations varied from 42,750 (S14) to 128,300 (S22) mg/kg, with an average value of 93,822 mg/kg. As shown in Table 2, approximately 83% of the Al concentrations are higher than the ECA of 80,500 mg/kg dw, while all Al concentrations are lower than the industrial soil average (ISA) of 355,588 mg/kg dw.^46,54^ The Fe concentrations in the soil samples varied from 57,700 (S18) to 149,700 (S14) mg/kg with an average value of 82,410 mg/kg. All Fe concentrations are higher than the ECA of 46,500 mg/kg dw^45^ and the ISA of 355,579 mg/kg dw.^54^ The Ti concentrations in the soil samples varied from 4796 (S18) to 11,800 (S14) mg/kg with an average value of 6623 mg/kg. All Ti concentrations are higher than the ECA of 4500 mg/kg dw.^46^ The Mn concentrations varied from 1168 (S18) to 2169 (S14) mg/kg, with an average value of 1642 mg/kg. All Mn concentrations are higher than the ECA of 1000 mg/kg dw and the ISA of 992 mg/kg dw.^46,54^ The *V* concentrations varied from 235 (S22) to 906 (S14) mg/kg with an average value of 406 mg/kg. All *V* concentrations are higher than the ECA of 90 mg/kg dw.^46^ The Zn concentrations varied from 83 (S16) to 2042 (S14) mg/kg, with an average value of 278 mg/kg. All Zn concentrations, except one sample, are higher than the ECA of 83 mg/kg dw, while approximately 61% and 13% of Zn concentrations are higher than the maximum contaminant level (MCL) of 150 mg/kg dw and the ISA of 248 mg/kg dw, respectively.^40,46,54^ The Cu concentrations varied from 33 (S14) to 981 (S12) mg/kg, with an average value of 207 mg/kg. Approximately 91% of Cu concentrations are higher than the ECA of 47 mg/kg and MCL of 50 mg/kg dw, while lower than ISA of 588 mg/kg dw.^40,46,54^ The Cr concentrations varied from 59 (S13) to 533 (S10) mg/kg, with an average value of 149 mg/kg. Approximately 70 and 57% of Cr concentrations are higher than the ECA of 83 mg/kg dw and the MCL of 100 mg/kg dw, respectively, while all Cr concentrations, except two soil samples, are lower than the ISA of 333 mg/kg dw.^40,46,54^ The Ni concentrations varied from 44 (S13) to 111 (S11) mg/kg, with an average value of 78 mg/kg. All and approximately 91% of Ni concentrations are higher than the MCL of 30 mg/kg dw and the ECA of 58 mg/kg dw, respectively, while all Ni concentrations are lower than the ISA of 125 mg/kg dw.^40,46,54^ The Pb concentrations varied from 18 (S16) to 338 (S11) mg/kg, with an average value of 68 mg/kg. All and approximately 43% of Pb concentrations are higher than the ECA of 16 mg/kg dw and the MCL of 50 mg/kg dw, respectively, while all Pb concentrations are lower than the ISA of 125 mg/kg dw, except for one soil sample.^40,46,54^ The Co concentrations varied from 13 (S18) to 42 (S14) mg/kg, with an average value of 32 mg/kg. Approximately 87% of Co concentrations are higher than the ECA of 18 mg/kg dw, MCL of 20 mg/kg dw, and ISA of 21 mg/kg dw.^40,46,54^ The As concentrations varied from 3 (S5) to 22 (S11) mg/kg, with an average value of 10 mg/kg. All and approximately 91% of As concentrations are lower than the MCL of 20 mg/kg dw and ISA of 500 mg/kg dw, respectively, while all As concentrations are higher than the ECA of 1.7 mg/kg dw.^40,46,54^

**Figure 4:**
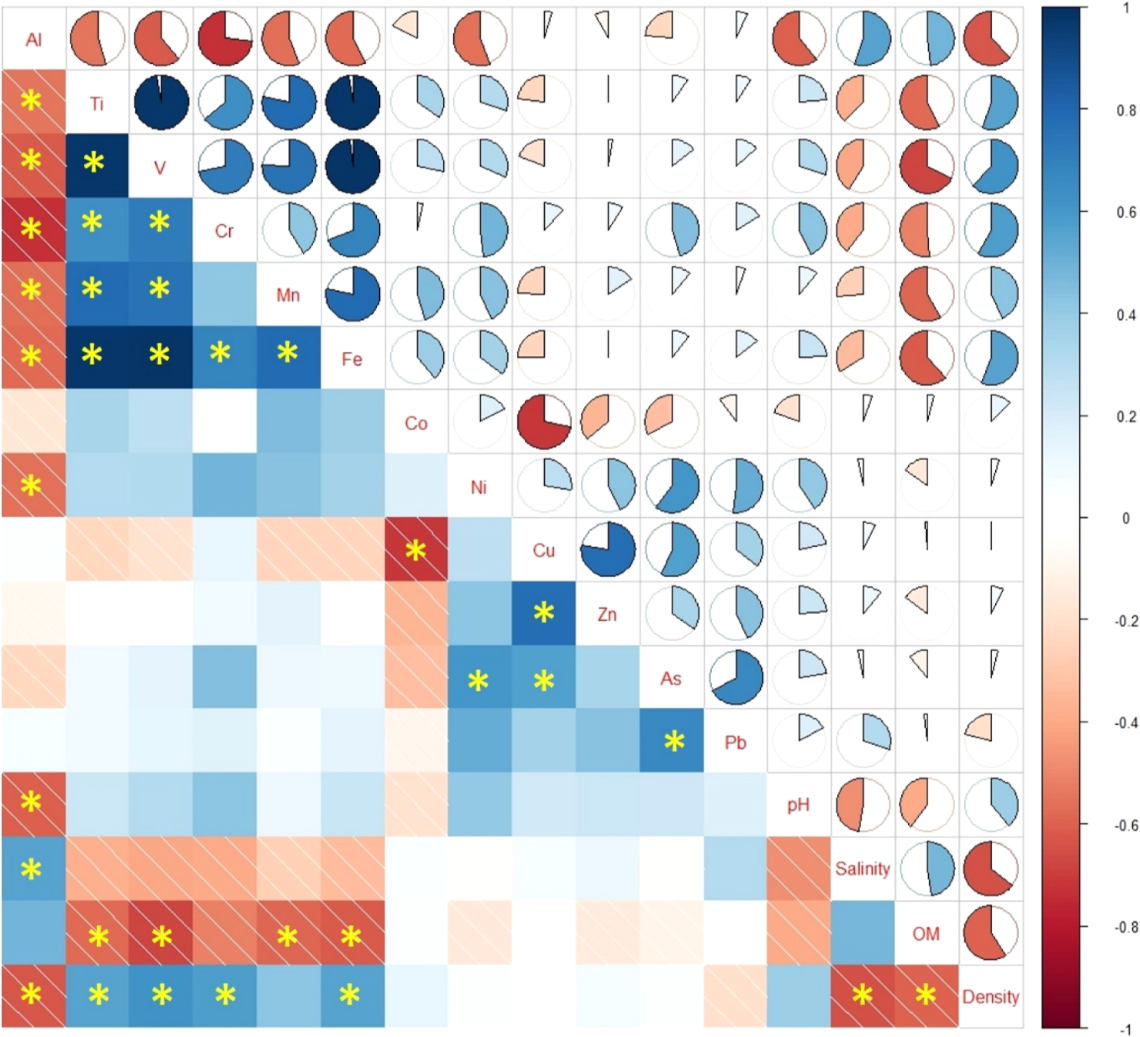
Correlogram plot for PTEs and physicochemical properties of topsoil samples. [*denotes a statistically significant correlation (*p* < 0.01)].

Pearson correlation analysis between the PTEs analyzed in the samples showed a significant correlation between some PTEs. A significant positive correlation (*p* ≤ 0.01) is found between Ti–V (0.97), Ti–Cr (0.64), Ti–Mn (0.78), Ti–Fe (0.98); V– Cr (0.72), V– Mn (0.76), V– Fe (0.98); Cr–Fe (0.69), Mn–Fe (0.78), Ni–As (0.60), Cu–Zn (0.78), Cu–As (0.56), and As–Pb (0.67). A positive correlation between PTEs in soil indicates that they probably have a common pollution source.^3^ There are also several negative correlations among some PTEs. Both significant positive and negative correlations can be seen in Figure 4.

In investigating the physicochemical properties of topsoil and their correlations with PTEs, notable positive and negative associations have emerged. Among these correlations, a particularly significant negative relationship was identified between the Al concentration and soil pH (−0.61). This negative correlation suggests that as soil pH decreases, which indicates increased acidity, the concentration of Al tends to rise. As Li and Johnson^55^ stated, Al solubility increases and, consequently, is more available in soil having acidic conditions. The correlation analysis also presented a positive correlation between the salinity and Al concentration. Gunasekera and Silva^56^ also revealed a positive relationship between soil salinity and Al concentration. They also reported that the concentration of Al was increased with rising soil salinity. In addition, significant negative correlations were observed between OM and Ti, V, Mn, and Fe. This negative correlation could be explained by the capacity of soluble OM to bind micropollutants, such as metals, and transport them to groundwater.^57^ Since OM can form complexes with OM, it decreases concentrations of some metals in the soil.

Density is known to be affected by several factors, and it is impacted by factors such as the OM in the soil, its texture, the composition of minerals, and porosity.^58^ Both the negative and positive correlations between soil density and some PTEs could be due to their contribution to soil porosity, leading to a change in soil density.

### 4.1 Ecological Risk Evaluation

The values of the PI, *I*_geo_, and EF estimated for PTEs in the analyzed soil samples are given in Table 3. The average values of PI decrease in the following order: As (5.83) > V (4.51) > Cu (4.41) > Pb (4.27) > Zn (3.35) > Cr (1.789) > Co (1.786) > Fe (1.77) > Mn (1.64) > Ti (1.47) > Ni (1.35) > Al (1.17). The average results reveal that the soil samples are very strongly polluted with As and strongly polluted with V, Cu, Pb, and Zn, while lowly polluted with Cr, Co, Fe, Mn, Ti, Ni, and Al. The PLI values of all PTEs calculated for the studied area varied from 0.6 to 3.5, with an average value of 1.6, indicating that the study area is contaminated with PTEs and the soil quality has deteriorated. The average values of *I*_geo_ decrease in the following order: As (1.77) > V (1.51) > Pb (1.12) > Cu (1.05) > Zn (0.65) > Co (0.204) > Fe (0.201) > Mn (0.11) > Cr (−0.01) > Ti (−0.06) > Ni (−0.18) > Al (−0.41). The average *I*_geo_ values indicate that the soil samples (i) are practically uncontaminated with Al, Ti, Cr, and Ni, (ii) are uncontaminated to moderately contaminated with Mn, Fe, Co, and Zn, and (iii) are moderately contaminated with As, Pb, Cu, and V. The average values of EF decrease in the following order: As (7.00) > V (5.42) > Cu (5.30) > Pb (5.12) > Zn (4.02) > Cr (2.150) > Co (2.145) > Fe (2.13) > Mn (1.97) > Ti (1.77) > Ni (1.62) > Al (1.40). The average EF values indicate that the soil samples are minimally enriched with Mn, Ti, Ni, and Al and moderately enriched with Zn, Cr, Co, and Fe, while significantly enriched with As, Pb, Cu, and V.

**Table 3:** Values of Single Pollution Index, Geo-Accumulation Index, and Enrichment Factor

PTE	value of index								
	PI			*I*_geo_			EF		
	average	min	max	average	min	max	average	min	max
Al	1.17	0.53	1.59	–0.41	–1.50	0.09	1.40	0.64	1.91
Ti	1.47	1.07	2.62	–0.06	–0.49	0.81	1.77	1.28	3.15
V	4.51	2.61	10.07	1.51	0.80	2.75	5.42	3.14	12.10
Cr	1.79	0.72	6.42	–0.01	–1.07	2.10	2.15	0.86	7.71
Mn	1.64	1.17	2.17	0.11	–0.36	0.53	1.97	1.40	2.61
Fe	1.77	1.24	3.22	0.20	–0.27	1.10	2.13	1.49	3.87
Co	1.79	0.73	2.33	0.20	–1.03	0.63	2.15	0.88	2.80
Ni	1.35	0.76	1.91	–0.18	–0.97	0.35	1.62	0.92	2.29
Cu	4.41	0.70	20.87	1.05	–1.09	3.80	5.30	0.85	25.08
Zn	3.35	1.00	24.60	0.65	–0.59	4.04	4.02	1.20	29.56
As	5.83	1.59	13.06	1.77	0.08	3.12	7.00	1.91	15.69
Pb	4.27	1.13	21.14	1.12	–0.41	3.82	5.12	1.36	25.40

### 4.2 Health Risk Evaluation

The values of the ADD, HQ, HI, ILCR, and CR estimated for PTEs analyzed in the investigated soil samples are summarized in Table 4. The ADD values estimated for adults varied from 1.3 × 10^–5^ (As) to 1.2 × 10^–1^ (Al) mg/kg/d. The average ADD values of the PTEs are ranked in the ascending order of As < Co < Pb < Ni < Cr < Cu < Zn < V < Mn < Ti < < Fe < Al. The HQ values varied from 0.0012 (Zn) to 1,502,894 (Fe).

**Table 4:** Noncarcinogenic and Carcinogenic Risks to Adults from PTEs in the Soil Samples

PTE		ADD (mg/kg day)	HQ	ILCR
Al	average	1.2 × 10^–1^	1.22	
	range	5.6 × 10^–2^ to 1.7 × 10^–1^	0.56–1.67	
Ti	average	8.6 × 10^–3^		
	range	6.2 × 10^–3^ to 1.5 × 10^–2^		
V	average	5.3 × 10^–4^	0.06	
	range	3.1 × 10^–4^ to 1.2 × 10^–3^	0.03–0.13	
Cr	average	1.9 × 10^–4^	0.06	9.6 × 10^–5^
	range	7.7 × 10^–5^ to 6.9 × 10^–4^	0.03–0.23	3.9 × 10^–5^ to 3.5 × 10^–4^
Mn	average	2.1 × 10^–3^	0.02	
	range	1.5 × 10^–3^ to 2.8 × 10^–3^	0.01–0.02	
Fe	average	1.1 × 10^–1^	15.3	
	range	7.5 × 10^–2^ to 1.9 × 10^–1^	10.71–27.77	
Co	average	4.2 × 10^–5^	0.14	
	range	1.7 × 10^–5^ to 5.4 × 10^–5^	0.06–0.18	
Ni	average	1.0 × 10^–4^	5.1 × 10^–3^	1.7 × 10^–4^
	range	5.8 × 10^–5^ to 1.4 × 10^–4^	2.9 × 10^–3^ to 7.2 × 10^–3^	9.8 × 10^–5^ to 2.4 × 10^–4^
Cu	average	2.7 × 10^–4^	5.4 × 10^–2^	
	range	4.3 × 10^–5^ to 1.3 × 10^–3^	8.6 × 10^–3^ to 2.5 × 10^–1^	
Zn	average	3.6 × 10^–4^	1.2 × 10^–3^	
	range	1.1 × 10^–4^ to 2.7 × 10^–3^	3.6 × 10^–4^ to 8.8 × 10^–3^	
As	average	1.3 × 10^–5^	4.3 × 10^–2^	1.9 × 10^–5^
	range	3.5 × 10^–6^ to 2.9 × 10^–5^	1.2 × 10^–2^ to 9.6 × 10^–2^	5.3 × 10^–6^ to 4.3 × 10^–5^
Pb	average	8.9 × 10^–5^	2.5 × 10^–2^	7.5 × 10^–7^
	range	2.4 × 10^–5^ to 4.4 × 10^–4^	6.7 × 10^–3^ to 1.3 × 10^–1^	2.0 × 10^–7^ to 3.7 × 10^–6^
HI = ΣHQ	average		16.9	
	range		12.3–28.9	
CR = ΣILCR	average			2.9 × 10^–4^
	range			1.5 × 10^–4^ to 5.9 × 10^–4^

The average HQ values of the PTEs are ranked as Zn < Ni < Mn < Pb < As < Cu < V < Cr < Co < Al < Fe. All HQ values are lower than the risk limit of 1, except for Al and Fe. The HI values varied from 12 to 19, with an average value of 17.

All HI values are higher than the risk limit of 1. The average ILCR estimated for Cr, Ni, As, and Pb were found as 9.6 × 10^–5^ (3.9 × 10^–5^ to 3.5 × 10^–4^), 1.7 × 10^–4^ (9.8 × 10^–5^ to 2.4 × 10^–4^), 1.9 × 10^–5^ (5.3 × 10^–6^ to 4.3 × 10^–5^), and 7.5 × 10^–7^ (2.0 × 10^–7^ to 3.7 × 10^–6^), respectively. The values for ILCR are ranked as follows: Ni > Cr > As > Pb. While the estimated ILCR values for As and Pb are in the safe range (10^–6^ to 10^–5^), approximately 74% of the ILCR values for Cr are in the low priority risk range (10^–5^ to 10^–4^). On the other hand, all ILCR values calculated for Ni, except one, exceeded the safety limit of 10^–4^. As a result, ILCR values reveal that the carcinogen risk from Ni is in an unacceptable risk range. The CR values estimated for Cr, Ni, As, and Pb varied from 1.5 × 10^–4^ to 5.9 × 10^–4^ with an average of 2.9 × 10^–4^. All CR values are above the safety limit.

## 5 Conclusions

In this study, the ecological and health risks caused by PTEs in soil samples from the Tekkeköy (Samsun) industrial zone, an important industrial region of Turkey, were evaluated in detail. The novel contribution of the study is that, for the first time, pollution in the industrial zone is evaluated from both ecological and health perspectives. Analysis results revealed that (1) the levels of PTEs (Al, Ti, V, Fe, Cr, Co, Cu, Mn, As, and Pb) analyzed in the investigated soil samples are greater than the ECA, and (2) the concentrations of Ni, Pb, Cr, Co, Cu, and Zn are also above the MCLs in the Turkish Regulation on Control of Soil Pollution. Ecological risk index values showed that the study area was strongly contaminated with As, Pb, Cu, and V. It is estimated that the contribution of the phosphogypsum waste area in this pollution is smaller than that of other industrial plants. As a result of the analysis, it was found that the concentrations of the mentioned PTEs contained in phosphogypsum were smaller than those contained in the soil samples.

All HI values are higher than the risk limit of 1. However, when the contributions of Al and Fe are subtracted from the HI values, all values are below the risk limit. This case indicates that exposure to Fe and Al poses the most significant noncarcinogenic potential health risk. All cancer risk values estimated for Cr, Ni, As, and Pb exceeded the safety limit. This case revealed that Ni and Cr contamination in soil samples is at risk for people living near the industrial zone.
